# Outcomes of solitary postoperative recurrence of esophageal squamous cell carcinoma diagnosed with FDG-PET/CT and treated with definitive radiation therapy

**DOI:** 10.1007/s10388-023-01000-4

**Published:** 2023-04-07

**Authors:** Hiroki Ihara, Kotaro Yoshio, Shunsuke Tanabe, Soichi Sugiyama, Masashi Hashimoto, Naoaki Maeda, Shinsuke Akagi, Soshi Takao, Kazuhiro Noma, Takao Hiraki

**Affiliations:** 1 Department of Radiology, Graduate School of Medicine, Dentistry and Pharmaceutical Sciences, Okayama University, 2-5-1 Shikata-cho, Kita-ku, Okayama, 700-8558 Japan; 2 Department of Proton Beam Therapy, Graduate School of Medicine, Dentistry and Pharmaceutical Sciences, Okayama University, 2-5-1 Shikata-cho, Kita-ku, Okayama, 700-8558 Japan; 3 Department of Gastroenterological Surgery, Graduate School of Medicine, Dentistry and Pharmaceutical Sciences, Okayama University, 2-5-1 Shikata-Cho, Kita-Ku, Okayama, 700-8558 Japan; 4 Department of Epidemiology, Graduate School of Medicine, Dentistry and Pharmaceutical Sciences, Okayama University, 2-5-1 Shikata-cho, Kita-ku, Okayama, 700-8558 Japan

**Keywords:** Radiation therapy, Esophageal squamous cell carcinoma, Recurrence, ^18^F-fluorodeoxyglucose positron emission tomography, Survival

## Abstract

**Background:**

Surgical resection of esophageal cancer is frequently performed to achieve a complete cure. However, the postoperative recurrence rate is 36.8–42.5%, leading to poor prognosis. Radiation therapy has been used to treat recurrences; solitary recurrence has been proposed as a prognostic factor for radiation therapy, though its significance is unclear. ^18^F-fluorodeoxyglucose positron emission tomography is a highly accurate diagnostic modality for esophageal cancer. This retrospective study aimed to analyze the outcomes of solitary postoperative recurrences of esophageal squamous cell carcinoma diagnosed with ^18^F-fluorodeoxyglucose positron emission tomography and treated with definitive radiation therapy.

**Methods:**

We examined 27 patients who underwent definitive radiation therapy for single or multiple postoperative recurrences of esophageal squamous cell carcinoma between May 2015 and April 2021. ^18^F-fluorodeoxyglucose positron emission tomography/computed tomography was performed within 3 months before the commencement of radiation therapy. Kaplan–Meier, univariate, and multivariate analyses were performed to examine the overall survival and identify potential prognostic factors.

**Results:**

The 1-, 2-, and 3-year overall survival rates were 85.2%, 62.6%, and 47.3%, respectively, and solitary recurrence was the only significant factor associated with overall survival (*P* = 0.003). The 1-, 2-, and 3-year overall survival rates in patients with solitary recurrence were 91.7%, 80.2%, and 80.2%, respectively, and in patients with multiple recurrences they were 80.0%, 50.3%, and 25.1%, respectively. Multivariate analysis also showed solitary recurrence as a significant factor for overall survival.

**Conclusions:**

When diagnosed with ^18^F-fluorodeoxyglucose positron emission tomography/computed tomography, solitary recurrence appears to have a more favorable prognosis than multiple recurrences.

## Introduction

Surgical resection of esophageal cancer is a regular treatment method to achieve a complete cure. However, postoperative recurrence rates range from 36.8 to 42.5%, leading to poor prognosis [1–3]. Therefore, it is crucial to treat recurrences, and radiation therapy is an adequate strategy for limited lesions. Several studies have reported the effectiveness of radiation therapy in esophageal cancer and investigated various prognostic factors for overall survival (OS) [4–20]. Recently, radiation therapy for oligometastatic cancers has received attention for various types of tumors [21], and solitary recurrence has been reported as a prognostic factor in esophageal cancer [4, 7, 10, 12, 14, 16, 18, 19]. However, these studies did not describe the frequency of use of ^18^F-fluorodeoxyglucose positron emission tomography (FDG-PET) for diagnosis before radiation therapy. Despite the high accuracy of FDG-PET in the initial diagnosis and recurrence of esophageal cancer [22–26], the impact of solitary recurrence diagnosed specifically using FDG-PET in radiation therapy has not been clarified.

We hypothesized that solitary recurrence, accurately diagnosed with FDG-PET before the radiation therapy, is an important factor for OS in esophageal cancer patients. This study aimed to analyze the outcomes of solitary recurrence of esophageal squamous cell carcinoma diagnosed using FDG-PET/computed tomography (CT) and treated with radiation therapy. To the best of our knowledge, no studies have evaluated the effect on survival after radiation therapy of solitary postoperative recurrence of esophageal cancer, specifically diagnosed with FDG-PET/CT, compared to multiple recurrences.

## Methods

This study was approved by the Institutional Review Board (Approval No. K2206-001) and followed the Declaration of Helsinki. The need for informed consent was waived because of the retrospective nature of the study. Data were collected from medical records and radiation therapy plans.

### Study population

We retrospectively analyzed patients treated with definitive radiation therapy for localized postoperative recurrent esophageal cancer between May 2015 and April 2021 at Okayama University Hospital. The initial surgery was basically radical subtotal esophagectomy with 2- or 3-field lymph node dissection. The inclusion criteria were: (1) primary tumor pathology confirmed as esophageal squamous cell carcinoma; (2) postoperative recurrent sites excluding the mucosa; (3) no history of radiation therapy for recurrent tumors; (4) no disseminated and/or hematogenous metastases, such as in the liver or lungs; (5) no other active cancers; (6) FDG-PET/CT performed within 3 months before initiation of radiation therapy; (7) all recurrent tumors scheduled for radiation of at least 50 Gy; and (8) at least one follow-up visit after radiation therapy completion. Patients who participated in esophageal cancer clinical trials were excluded.

Clinical stages were determined based on the 8^th^ Edition of the Union for International Cancer Control TNM classification. Recurrence was diagnosed comprehensively by surgeons and radiation oncologists using physical findings, tumor markers, endoscopy, CT, and FDG-PET/CT findings.

### Treatment

Radiation therapy was performed using X-ray beams 5 days/week. The prescribed dose was 50–66 Gy in fractions of 1.8 or 2.0 Gy. The typical radiation dose was 60 Gy in fractions of 2.0 Gy. When the reconstructed intestinal tract and/or small bowel was irradiated, a dose of 50–54 Gy in fractions of 1.8 or 2.0 Gy was selected. CT simulation was used. Gross tumor volume (GTV) was determined using CT and FDG-PET/CT. The clinical target volume (CTV) was defined as GTV plus a 0.5–1 cm margin. The planning target volume was defined as CTV plus a margin of 0.5 cm. An internal margin was also considered if the tumor moved with the respiration. Three-dimensional conformal radiation therapy (3D-CRT) was used in most cases. Intensity-modulated radiation therapy (IMRT) was considered if the irradiated area included the neck. Surgeons and radiation oncologists determined whether prophylactic irradiation was required. Systemic chemotherapy was administered concurrently if possible. Surgeons used performance status, treatment history, and other factors to determine chemotherapy regimens.

### Follow-up

Follow-up was conducted approximately at 3-month intervals for the first 2 years after radiation therapy and every 6 months thereafter. Recurrence was evaluated using physical findings, tumor markers, endoscopy, CT, magnetic resonance imaging, and FDG-PET/CT findings.

Late adverse events were defined as those that emerged ≥ 91 days after initiation of radiation therapy and were evaluated using the Common Terminology Criteria for Adverse Events, version 5.0. Only non-hematologic adverse events of grade > 2 were investigated.

### Prognostic factors

Several possible prognostic factors were evaluated, including sex, age, performance status, initial clinical stage, number of tumors, tumor diameter, location of recurrence, interval from final surgery to diagnosis of recurrence, radiation dose, prophylactic irradiation, and concurrent chemotherapy. Age and performance status were assessed at the beginning of the radiation therapy. Performance status was evaluated using the Eastern Cooperative Oncology Group (ECOG) scale. Based on the tumor number, patients were categorized into solitary or multiple tumor groups. Tumor diameter was defined as the long-axis diameter; if multiple tumors were present, the diameter of the largest tumor was considered as the tumor diameter. The minimum prescribed dose was selected if multiple tumors were treated with different doses.

### Statistical analyses

Kaplan–Meier analyses were performed to determine OS, progression-free survival (PFS), and local control (LC) rates starting from initiation of radiation therapy until death from cancer or other causes, disease progression or death from cancer or other causes, and tumor progression within the irradiation field, respectively.

Cutoff values to categorize factors such as age, recurrent tumor diameter, interval from final surgery to diagnosis of recurrence, and radiation dose were determined according to the median values of the study population. Univariate analyses of various potential prognostic factors for OS, PFS, and LC were performed using log-rank tests.

If the number of tumors was revealed as a significant prognostic factor in univariate analysis, the solitary and multiple recurrence groups were compared using two-sided Fisher’s exact test for categorical variables. Furthermore, we estimated hazard ratios (HRs) and 95% confidence intervals (CIs) for OS using the Cox proportional hazard model to adjust for potential confounders in the multivariate analysis. Various factors were sequentially included in three models: we started by analyzing a crude model (model 1, including only the number of tumors), then we adjusted for sex and age (model 2). Subsequently, we adjusted the analysis for factors that were significantly different between the solitary and multiple recurrence groups and for significant risk factors in the log-rank test (model 3).

*P* values < 0.05 indicated statistically significant differences. In the crude and multivariate analyses of OS, HRs > 1.00 indicated an increased risk of death.

Kaplan–Meier and Cox proportional hazard model-based analyses were performed using the IBM Statistical Package for the Social Sciences for Windows, version 26 (IBM, Armonk, NY, USA). Fisher’s exact test was performed using Stata 17 software (StataCorp LP, College Station, TX, USA).

## Results

### Patient and treatment characteristics

Patient and treatment characteristics are presented in Table 1. In total, 27 patients were included in the analysis (24 males and 3 females; median age: 70 years, range 49–86). A solitary lesion was diagnosed in 12 patients, whereas 15 presented ≥ 2. The median tumor diameter was 29 mm (range 12–49). The median follow-up time was 24 months (range 5–71). Regarding the initial clinical stage, 4, 10, 4, and 9 patients were in stages I, II, III, and IV, respectively. Patients with stage IV disease had no distant metastases other than supraclavicular lymph nodes. Before the initial surgery, 22 patients underwent chemotherapy. Seven patients underwent surgery for the first postoperative recurrence. One patient underwent additional surgery for the second postoperative recurrence. Therefore, they underwent radiation therapy for the second and third recurrence after surgery. All patients completed the planned radiation therapy. Moreover, systemic chemotherapy was administered simultaneously in 25 patients; the remaining two were treated solely with radiation therapy because of advanced age or renal failure. Concurrently used regimens included tegafur/gimeracil/oteracil potassium in 18 patients; cetuximab in 2; cisplatin, docetaxel, and 5-fluorouracil in 2; cisplatin and 5-fluorouracil in 1; cisplatin in 1; and docetaxel in 1. The median radiation dose in 27 patients was 60 Gy (50–66 Gy). Furthermore, 22 patients received a dose of ≥ 60 Gy. Two patients were treated with IMRT, and the other 25 with 3D-CRT. All 12 patients with solitary recurrence were treated with concurrent chemoradiotherapy and received a radiation dose of 60 Gy, without prophylactic irradiation. The initial clinical stage and radiation dose were significantly different between the solitary and multiple recurrence groups (*P* = 0.006 and *P* = 0.047, respectively).

**Table 1:** Patient and treatment characteristics

	No. of patients
Sex	
Male/female	24/3
Age (years), (median 70; range 49–86)	
< 70/ ≥ 70	11/16
Performance status	
0/1	7/20
Initial clinical stage	
I/II/III/IV	4/10/4/9
Number of tumors	
1/2/3/4/ ≥ 5	12/6/2/3/4
Tumor diameter (mm), (median 29; range 12–49)	
< 29/ ≥ 29	13/14
Location of recurrence	
Mediastinum/abdomen/mediastinum and neck/mediastinum and abdomen/mediastinum and pulmonary hilum	16/4/5/1/1
Interval from final surgery to diagnosis of recurrence (months), (median 7; range 1–31)	
< 7/ ≥ 7	12/15
Radiation dose (Gy)	
< 60/ ≥ 60	5/22
Prophylactic irradiation	
Yes/no	4/23
Concurrent chemotherapy	
Yes/no	25/2

### Treatment outcome

**Fig. 1:**
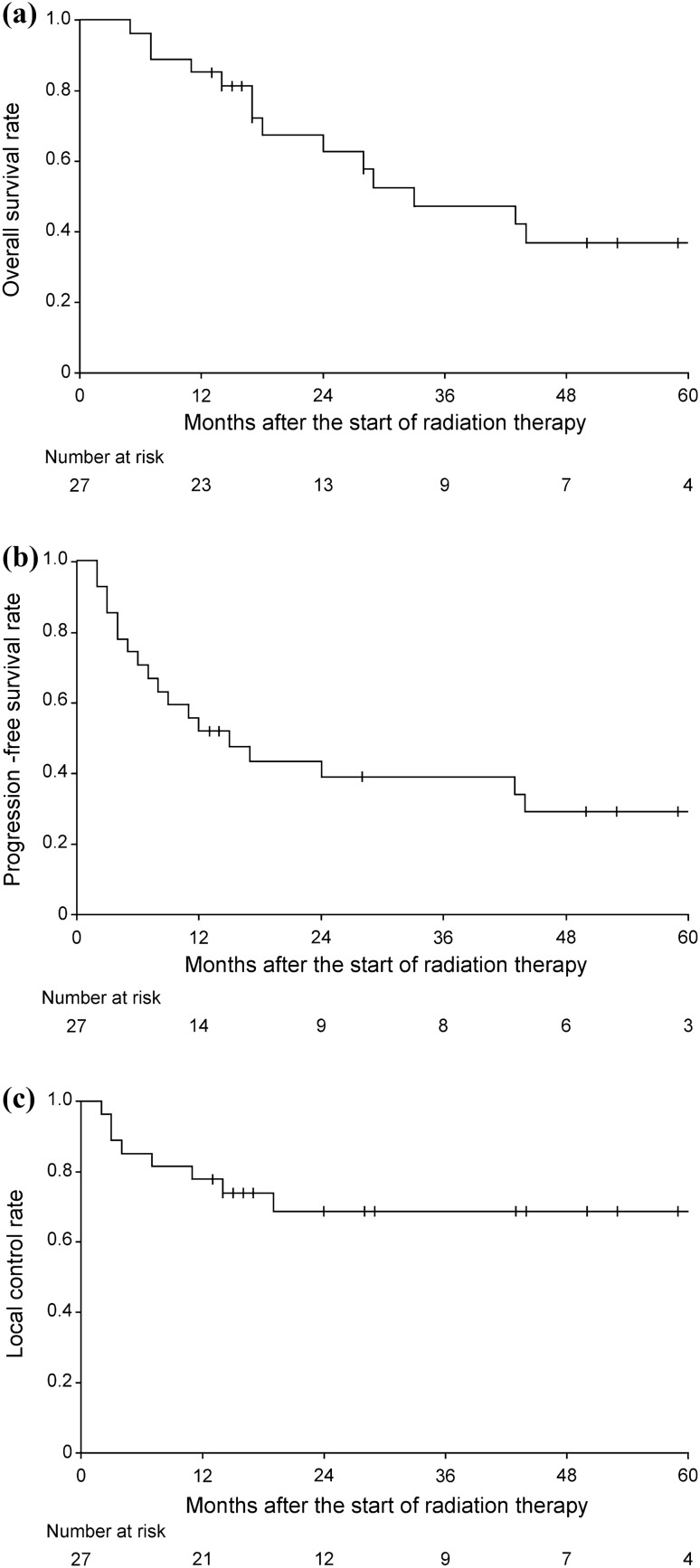
Kaplan–Meier curves for all patients. **a** Overall survival; **b** progression-free survival; **c** local control rate

The 1-, 2-, and 3-year OS rates were 85.2%, 62.6%, and 47.3%, respectively (median OS: 33 months); PFS rates were 51.9%, 38.9%, and 38.9%, respectively (median PFS: 15 months); and LC rates were 77.8%, 68.6%, and 68.6%, respectively (Fig. 1). In-field and out-of-field recurrences occurred in 8 and 14 patients, respectively. Seven patients had both in-field and out-of-field recurrences, and one patient had only in-field recurrence. In-field recurrences occurred between 2 and 19 months (median 5.5) after initiation of radiation therapy. Six patients underwent additional radical treatment for localized recurrences: three underwent surgery and three underwent radiation therapy. Unfortunately, 15/27 patients died. No late non-hematologic adverse events of grade > 2 were observed during the follow-up period.

**Fig. 2:**
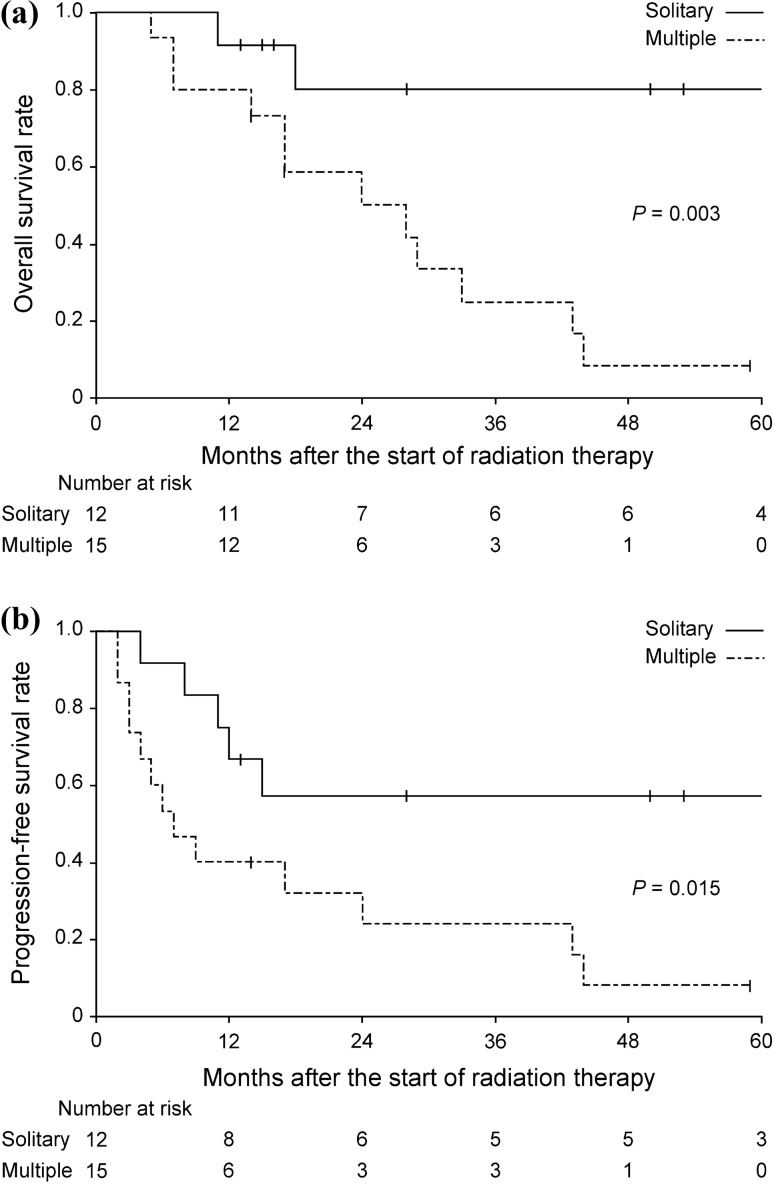
Kaplan–Meier curves for patients with solitary recurrence (solid line) or multiple recurrences (dashed line). **a** Overall survival; **b** progression‑free survival rate

In the univariate analysis of all variables considered, only solitary recurrence was a significant prognostic factor for OS and PFS (*P* = 0.003 and *P* = 0.015, respectively; Fig. 2). The 1-, 2-, and 3-year OS rates of patients with solitary recurrence were 91.7%, 80.2%, and 80.2%, respectively, whereas those of patients with multiple recurrences were 80.0%, 50.3%, and 25.1%, respectively. In contrast, only the interval from final surgery to diagnosis of recurrence was a significant prognostic factor for LC (*P* = 0.036). Results of the univariate analysis for each variable considered are presented in Table 2.

**Table 2:** Univariate analysis of overall survival, progression‑free survival, and local control

	No. of patients		Overall survival			Progression-free survival			Local control		
			2-year (%)		*P*	2-year (%)		*P*	2-year (%)		*P*
Sex											
Male	24		62.7		0.827	35.0		0.724	68.5		0.980
Female	3		66.7			66.7			66.7		
Age (years)											
< 70	11		70.1		0.601	32.7		0.925	63.6		0.514
≥ 70	16		58.7			42.9			72.2		
Performance status											
0	7		47.6		0.431	21.4		0.358	71.4		0.983
1	20		67.5			44.0			68.4		
Initial clinical stage											
I, II	14		68.1		0.266	41.7		0.381	68.8		0.797
III, IV	13		56.1			35.9			69.2		
Number of tumors											
Solitary	12		80.2		0.003**	57.1		0.015*	83.3		0.201
Multiple	15		50.3			24.0			57.1		
Tumor diameter (mm)											
< 29	13		84.6		0.082	60.6		0.105	84.6		0.101
≥ 29	14		39.3			17.9			51.4		
Interval from final surgery to diagnosis of recurrence (months)											
< 7	12		57.1		0.367	31.3		0.342	46.7		0.036*
≥ 7	15		67.5			45.0			86.7		
Radiation dose (Gy)											
< 60	5		80.0		0.803	20.0		0.380	40.0		0.174
≥ 60	22		58.3			43.6			77.3		
Prophylactic irradiation											
Yes	4		25.0		0.451	25.0		0.383	50.0		0.213
No	23		70.6			41.1			72.0		
Concurrent chemotherapy											
Yes	25		64.5		0.498	38.4		0.965	65.8		0.366
No	2		50.0			50.0			100.0		

*P < 0.05; **P < 0.01

In the multivariate analysis, the number of tumors, sex, age, initial clinical stage, radiation dose, and interval from final surgery to diagnosis of recurrence were entered into the Cox proportional hazards model for model 3. Solitary recurrence was statistically significant in model 1 (HR 0.142; 95% CI 0.031–0.646), model 2 (HR 0.142; 95% CI 0.031–0.658), and model 3 (HR 0.031; 95% CI 0.004–0.274). Results of the multivariate analysis for OS are presented in Table 3.

**Table 3:** Multivariate analysis of overall survival

	Case/total number (%)	Model 1		Model 2			Model 3	
		HR	(95% CI)	HR		(95% CI)	HR	(95% CI)
Number of tumors								
Solitary (vs. multiple^a^)	12/27 (44.4)	0.142	(0.031–0.646)*	0.142		(0.031–0.658)*	0.031	(0.004–0.274)*
Sex								
Female (vs. male^a^)	3/27 (11.1)			1.164		(0.240–5.640)	1.056	(0.198–5.632)
Age (years)								
≥ 70 (vs. < 70^a^)	16/27 (59.3)			1.003		(0.309–3.253)	1.408	(0.415–4.769)
Initial clinical stage								
I, II (vs. III, IV^a^)	14/27 (51.9)						3.406	(0.673–17.237)
Radiation dose (Gy)								
≥ 60 (vs. < 60^a^)	22/27 (81.5)						8.677	(0.963–78.164)
Interval from final surgery to diagnosis of recurrence (months)								
< 7 (vs. ≥ 7^a^)	12/27 (44.4)						3.549	(0.917–13.743)

HR hazard ratio, CI confidence interval

^a^Reference category

*Statistically significant. Model 1: based only on number of tumors; model 2: analysis adjusted for sex and age; model 3: analysis adjusted also for initial clinical stage, radiation dose, and interval from surgery to diagnosis of recurrence

## Discussion

Radiation therapy or surgery are the main treatment options in postoperative recurrence of esophageal squamous cell carcinoma, if the sites of recurrence are limited. Nakamura et al. reported that patients with postoperative recurrent lymph node metastasis who underwent lymphadenectomy and chemoradiotherapy showed significantly higher survival rates than patients who received only chemotherapy or best supportive care [16]. Multimodal treatments, including lymphadenectomy and chemoradiotherapy, could improve survival in patients with esophageal carcinoma lymph node recurrence after curative resection.

Several studies have examined the effectiveness of radiation therapy for postoperative recurrent esophageal carcinoma [4–20], and the 2-year OS rates of radiation therapy vary between 15 and 78% [5, 6, 8, 10, 11, 13, 17, 20]. Numerous prognostic factors for the outcome after radiation therapy were reported, including age [6, 20], performance status [9, 13, 17], tumor size [8–10, 12, 17], number of recurrences [4, 7, 10, 12], disease-free interval [4, 9, 20], total dose [4, 17], concurrent chemotherapy [9, 12, 15]. We focused particularly on the number of recurrences, among all potential prognostic factors, because the definitive treatment of oligometastatic cancers has received significant attention recently. Palma et al. described a randomized, phase 2, open-label trial for stereotactic ablative radiotherapy (SABR) for various types of oligometastatic cancers, including breast, colorectal, lung, and prostate cancer [21]. SABR was associated with improved OS.

Regarding esophageal cancer, solitary recurrence after curative treatment was investigated in several studies including not only radiation therapy, but also other treatment options [4, 5, 7–10, 12, 14, 16–19]. Moreover, some studies showed that solitary recurrence was a favorable prognostic factor for OS [4, 7, 10, 12, 14, 16, 18, 19]. In our analyses, solitary recurrence was the only significant positive prognostic factor for OS. Chu et al. analyzed radiation therapy for cervical lymph node recurrence in thoracic esophageal squamous cell carcinoma after curative resection [4]. Univariate and multivariate analyses showed single lymph node recurrence as a favorable prognosis factor. Kawamoto et al. investigated the prognostic factors regarding chemoradiotherapy for postoperative lymph node recurrences of esophageal squamous cell carcinoma [7]. Univariate analysis showed that single recurrence was associated with significantly better prognosis. Ma et al. analyzed the effect of radiation therapy on recurrent mediastinal lymph node metastases and reported that the number of locoregional recurrences of these metastases (= 1 vs. > 1) was a prognostic factor in multivariate analysis [12]. However, other studies showed that solitary recurrence was not a significant prognostic factor [5, 9, 17]; we hypothesized that it was not significant because FDG-PET was not performed consistently before radiation therapy. Furthermore, none of these studies on solitary recurrence described the frequency of FDG-PET use before radiation therapy. With other imaging modalities, the conclusive diagnosis of solitary recurrence may have been less accurate. In this study, we might have been able to evaluate true solitary recurrences because all patients were evaluated using FDG-PET/CT.

The usefulness of FDG-PET/CT in the initial diagnosis of esophageal cancer has been reported [22, 23]. Additionally, several studies have also suggested its efficacy for follow-up and monitoring after surgery [24–26]. Kudou et al. reported that FDG-PET/CT has a high capability to detect single small recurrent tumors even outside the chest and abdomen and proposed a follow-up method using FDG-PET/CT after esophageal cancer surgery [24]. Pande et al. evaluated the diagnostic performance of FDG-PET/CT in the suspected recurrence of esophageal carcinoma after surgical resection with curative intent [25]. The sensitivity, specificity, and positive and negative predictive values of FDG-PET/CT were 98.4%, 80%, 98%, and 80%, respectively, with an accuracy of 97%. Based on the evidence of distant metastases identified on FDG-PET/CT, a change in management—from radiation therapy/surgery to palliative chemotherapy/best supportive care—was adopted in 41% (28/68) of patients. Furthermore, Goense et al. reported that in particular, FDG-PET and FDG-PET/CT show a minimal false-negative rate [26]. Pooled estimates of sensitivity and specificity for FDG-PET and FDG-PET/CT in diagnosing recurrent esophageal cancer were 96% and 78%, respectively.

In our analyses, the 1-, 2-, and 3-year OS rates overall were 85.2%, 62.6%, and 47.3%, respectively. Kawamoto et al. retrospectively evaluated 21 patients with postoperative solitary lymph node recurrence of esophageal squamous cell carcinoma [8]. Solitary lymph node recurrence was defined as follows: (1) ultrasonography, CT, and physical findings showed single lymph node and (2) PET showed focal uptake at the same lymph node. The median follow-up period was 32 months. The 2-year OS rate was 78%. The OS rates in our study and Kawamoto's study are high compared to those reported in previous studies [4–7, 9–20], possibly due to FDG-PET/CT aiding in appropriate patient selection and GTV description. However, possible false-positive cases must be carefully considered. Goense et al. emphasized that histopathological confirmation of a lesion suspected with FDG-PET or FDG-PET/CT is required owing to a considerable false-positive rate [26]. We also agree that a histopathological diagnosis should be performed if the imaging diagnosis is unclear. Nonetheless, FDG-PET is a crucial modality for judging the extent of the tumor; therefore, FDG-PET should be conducted before radiation therapy for postoperative recurrent esophageal squamous cell carcinoma.

The 1-, 2-, and 3-year LC rates were 77.8%, 68.6%, and 68.6%, respectively, in our study. In-field recurrence occurred in eight patients with an unsatisfactory LC rate. When the LC rates improve, the OS rates may also improve. Various techniques have been developed for radiation therapy; Liu et al. reported a phase 2 study of stereotactic body radiation therapy for patients with oligometastatic esophageal squamous cell carcinoma [5]. The median follow-up time was 18.2 months, and the 1- and 2-year OS rates were 76.2% and 58.0%, respectively. Furthermore, the 1- and 2-year LC rates were impressive, both 92.1%. In addition, Ishikawa et al. described a case report with successful proton-beam therapy [27]. Currently, the optimal technique for radiation therapy has yet to be established. Moreover, the efficacy of the combination of radio- and chemotherapy is yet to be clarified. In our study, the interval from final surgery to diagnosis of recurrence was the only significant prognostic factor for LC. When the interval is longer, the recurrent tumor is likely to be growing slowly. We hypothesized that this aspect could explain why the interval was a significant prognostic factor. Although the disease-free interval has been reported as a prognostic factor for OS [4, 9, 20], its impact on the LC has yet to be investigated.

This study has limitations. First, it was a retrospective study conducted at a single institution; therefore, the relatively short median follow-up period of 24 months may have been insufficient to evaluate the impact of the factors considered on long-term survival and late adverse events. Second, the sample size was limited, and no recurrent lesions were pathologically confirmed. Furthermore, not all patients received concurrent chemotherapy, and the regimens were inhomogeneous; the treatment strategy after radiation therapy varied with patient situations. Currently, immunotherapy is considered as a promising strategy, including nivolumab [28]; in our study, this medication was administered to four patients after failed radiation therapy, possibly contributing to their survival.

In conclusion, solitary recurrence appears to have a more favorable prognosis than multiple recurrences, when diagnosed using FDG-PET/CT. Further prospective multicenter studies are required to validate our findings and determine the optimal treatment strategy for postoperative localized recurrence in esophageal cancer. Additionally, more intensive radiation therapy and combination therapy might need to be considered in cases of solitary recurrence to improve prognosis. 

## Data Availability

The data that support the findings of this study are available from the corresponding author upon reasonable request.

## References

[1] Miyata H, Yamasaki M, Kurokawa Y (2011). Survival factors in patients with recurrence after curative resection of esophageal squamous cell carcinomas. Ann Surg Oncol.

[2] Toh Y, Oki E, Minami K (2010). Follow-up and recurrence after a curative esophagectomy for patients with esophageal cancer: the first indicators for recurrence and their prognostic values. Esophagus.

[3] Kato H, Fukuchi M, Miyazaki T (2005). Classification of recurrent esophageal cancer after radical esophagectomy with two- or three-field lymphadenectomy. Anticancer Res.

[4] Chu J, Wang F (2023). Prognostic analysis of radiotherapy for cervical lymph node recurrence after curative resection of the thoracic esophageal squamous cell carcinoma. J Radiat Res.

[5] Liu Q, Zhu Z, Chen Y (2020). Phase 2 study of stereotactic body radiation therapy for patients with oligometastatic esophageal squamous cell carcinoma. Int J Radiat Oncol Biol Phys.

[6] Chen J, Yin W, Yao H (2019). Salvage treatment for lymph node recurrence after radical resection of esophageal squamous cell carcinoma. Radiat Oncol.

[7] Kawamoto T, Nihei K, Sasai K (2018). Clinical outcomes and prognostic factors of chemoradiotherapy for postoperative lymph node recurrence of esophageal cancer. Jpn J Clin Oncol.

[8] Kawamoto T, Nihei K, Sasai K (2018). Involved-field chemoradiotherapy for postoperative solitary lymph node recurrence of esophageal cancer. Esophagus.

[9] Yamashita H, Jingu K, Niibe Y (2017). Definitive salvage radiation therapy and chemoradiation therapy for lymph node oligo-recurrence of esophageal cancer: a Japanese multi-institutional study of 237 patients. Radiat Oncol.

[10] Kimoto T, Yamazaki H, Suzuki G (2017). Local field radiotherapy without elective nodal irradiation for postoperative loco-regional recurrence of esophageal cancer. Jpn J Clin Oncol.

[11] Kobayashi R, Yamashita H, Okuma K (2014). Salvage radiation therapy and chemoradiation therapy for postoperative locoregional recurrence of esophageal cancer. Dis Esophagus.

[12] Ma DY, Tan BX, Liu M (2014). Concurrent three-dimensional conformal radiotherapy and chemotherapy for postoperative recurrence of mediastinal lymph node metastases in patients with esophageal squamous cell carcinoma: a phase 2 single-institution study. Radiat Oncol.

[13] Jingu K, Matsushita H, Takeda K (2012). Long-term results of radiotherapy combined with nedaplatin and 5-fluorouracil for postoperative loco-regional recurrent esophageal cancer: update on a phase II study. BMC Cancer.

[14] Kosuga T, Shiozaki A, Fujiwara H (2011). Treatment outcome and prognosis of patients with lymph node recurrence of thoracic esophageal squamous cell carcinoma after curative resection. World J Surg.

[15] Lu JC, Kong C, Tao H (2010). Radiotherapy with or without concurrent chemotherapy for lymph node recurrence after radical surgery of thoracic esophageal squamous cell carcinoma. Int J Radiat Oncol Biol Phys.

[16] Nakamura T, Ota M, Narumiya K (2008). Multimodal treatment for lymph node recurrence of esophageal carcinoma after curative resection. Ann Surg Oncol.

[17] Shioyama Y, Nakamura K, Ohga S (2007). Radiation therapy for recurrent esophageal cancer after surgery: clinical results and prognostic factors. Jpn J Clin Oncol.

[18] Yano M, Takachi K, Doki Y (2006). Prognosis of patients who develop cervical lymph node recurrence following curative resection for thoracic esophageal cancer. Dis Esophagus.

[19] Shimada H, Kitabayashi H, Nabeya Y (2003). Treatment response and prognosis of patients after recurrence of esophageal cancer. Surgery.

[20] Nemoto K, Ariga H, Kakuto Y (2001). Radiation therapy for loco-regionally recurrent esophageal cancer after surgery. Radiother Oncol.

[21] Palma DA, Olson R, Harrow S (2019). Stereotactic ablative radiotherapy versus standard of care palliative treatment in patients with oligometastatic cancers (SABR-COMET): a randomised, phase 2, open-label trial. Lancet.

[22] Kato H, Miyazaki T, Nakajima M (2005). The incremental effect of positron emission tomography on diagnostic accuracy in the initial staging of esophageal carcinoma. Cancer.

[23] van Westreenen HL, Westerterp M, Bossuyt PMM (2004). Systematic review of the staging performance of 18F-fluorodeoxyglucose positron emission tomography in esophageal cancer. J Clin Oncol.

[24] Kudou M, Shiozaki A, Fujiwara H (2016). Efficacy of PET-CT in the diagnosis and treatment of recurrence after esophageal cancer surgery. Anticancer Res.

[25] Pande SS, Purandare N, Puranik A (2020). Role of 18F-FDG PET/CT in restaging of esophageal cancer after curative-intent surgical resection. Nucl Med Commun.

[26] Goense L, van Rossum PSN, Reitsma JB (2015). Diagnostic performance of ^18^F-FDG PET and PET/CT for the detection of recurrent esophageal cancer after treatment with curative intent: a systematic review and meta-analysis. J Nucl Med.

[27] Ishikawa Y, Suzuki M, Yamaguchi H (2022). Long-term survival after definitive proton beam therapy for oligorecurrent esophageal squamous cell carcinoma: a case report. J Med Case Rep.

[28] Kudo T, Hamamoto Y, Kato K (2017). Nivolumab treatment for oesophageal squamous-cell carcinoma: an open-label, multicentre, phase 2 trial. Lancet Oncol.

